# Assessing the impact of COVID-19 interventions on the hand, foot and mouth disease in Guangdong Province, China: a Bayesian modeling study

**DOI:** 10.3389/fpubh.2023.1307321

**Published:** 2024-01-11

**Authors:** Li Zhang, Fen Yang, Zhihua Zhu, Weilin Zeng, Zuhua Rong, Jianxiong Hu, Xing Li, Jianguo Zhao, Biao Zeng, Yihan Li, Yi Quan, Qian Zhang, Zitong Huang, Yuye Li, Xing Huang, Wenyuan Zheng, Jiaqing Xu, Yan Li, Qing Chen, Jianpeng Xiao, Meng Zhang

**Affiliations:** ^1^School of Public Health, Southern Medical University, Guangzhou, China; ^2^Guangdong Provincial Institute of Public Health, Guangdong Provincial Center for Disease Control and Prevention, Guangzhou, China; ^3^Guangdong Provincial Center for Disease Control and Prevention, Guangzhou, China; ^4^Guangdong Workstation for Emerging Infectious Disease Control and Prevention, Guangzhou, China; ^5^School of Public Health, Guangdong Pharmaceutical University, Guangzhou, China; ^6^School of Public Health, Sun Yat-sen University, Guangzhou, Guangdong, China; ^7^School of Medicine, Jinan University, Guangzhou, China

**Keywords:** HFMD, COVID-19, SARS-CoV-2, non-pharmaceutical interventions, impact, BSTS

## Abstract

**Background:**

The non-pharmaceutical interventions (NPIs) against COVID-19 may have affected the transmission of hand, foot and mouth disease (HFMD). We aimed to assess the impact of the NPIs on HFMD in the high epidemic area of HFMD, Guangdong Province.

**Methods:**

The data of HFMD cases, etiological information, and meteorological factors in Guangdong from January 1, 2012, to December 31, 2021, were collected. Using a Bayesian structural time series (BSTS) model integrated counterfactual framework, we assessed the effect of NPIs on HFMD by different intervention periods, populations (gender, age, occupation), and cities. We further explored the correlation between the reduction of HFMD and socioeconomic factors in 21 cities.

**Results:**

A total of 351,217 HFMD cases were reported and 455,327 cases were averted in Guangdong Province during 2020–2021 with a reduction of 84.94% (95%CI: 81.63–87.22%) in 2020 and 29.49% (95%CI: 15.26–39.54%) in 2021. The impact of NPIs on HFMD differed by age and gender. The effects of NPIs were more remarkable for children aged 0–2 years and scattered children. We found that the relative reductions in 21 cities were related to the composition ratio of children and COVID-19 incidence.

**Conclusion:**

The reduction of HFMD incidence was significantly associated with COVID-19 NPIs, and school closure was an effective intervention to prevent HFMD outbreaks. Our findings will contribute to the development of HFMD prevention and control measures.

## 1 Introduction

Hand, foot and mouth disease (HFMD) is a widespread infectious disease that mainly affects children (1). The average annual number of HFMD cases reported in China from 2014 to 2019 was approximately 2.0 million (2). Guangdong has historically been a region with a high incidence of HFMD (3). The incidence of HFMD was about three times the national average, making it a major public health concern (4, 5). It has been reported that HFMD in China had semiannual peaks of activity, including a major peak in summer and a smaller peak in autumn (6). The incidence of HFMD remains high, threatening the health of children and causing a significant burden in China.

Coronavirus disease 2019 (COVID-19) which caused by SARS-CoV-2 is highly infectious (7). During the COVID-19 epidemic, China has adopted a series of non-pharmaceutical interventions (NPIs), such as school closure, wearing masks and enhancing personal hand hygiene, to control the spread of COVID-19 (8). Several studies have shown that the NPIs against the COVID-19 epidemic have co-benefits for the prevention of other infectious diseases. For example, the respiratory diseases decreased from 156.24 per 100,000 people during 2014–2019 to 145.12 in 2020 (9). Geng et al. found that the incidence of pertussis decreased by 76.49% in 2020, compared to the average incidence in 2017–2019 (10). Additionally, Zhao et al. showed that HFMD cases were reduced by 52.9% in 2020 (11). A study in Xi’an City found that HFMD cases decreased by 94.2% in the first half of 2020 (12). Xiao et al. found that HFMD has decreased by 83.9% compared to the average incidence in Guangdong Province during the anti-COVID-19 period (13). It was reported that the HFMD cases in China increased again in 2021, with reported cases of 1.35 million (vs. 0.76 million in 2020) (14, 15). Li′s study found that the incidence of HFMD in 2021 has higher than expected (12). However, there was less knowledge about the impact on HFMD with the NPIs during the different periods of COVID-19 epidemic.

In this study, based on large population surveillance data in the high epidemic area of HFMD, Guangdong Province, China, we aim to investigate the changing epidemic characteristics of HFMD in 2020–2021, the first two years during the COVID-19 pandemic. Then we quantified the effects of COVID-19 interventions on the HFMD epidemic in 2020–2021.

## 2 Methods

### 2.1 Study area and participants

Guangdong Province is located between 20°09′–25°31’ N latitude and 109°45′–117°20′ E longitude (16) in southern China, including 21 cities, with a population of 12.6 billion (17). It belongs to the East Asian monsoon region, with hot and rainy seasons (18). Guangdong is the province with the largest number of reported HFMD cases (19), ranking first among notifiable infectious diseases from 2010 to 2018 (20), and the cases were 418,300 in 2019, accounting for 21.9% of China (21, 22). HFMD has decreased significantly in the initial phase of COVID-19 epidemic (13), for the reason that Guangdong implemented strict NPIs such as travel restrictions, school closure, compulsory health quarantine, and regional lockdown. Thus, Guangdong would be an ideal site to study the effects of COVID-19 intervention on HFMD. Supplementary Figure S1 shows the distribution of 21 cities and populations in Guangdong Province in 2020. In this study, the study population was children aged 0–14 years.

### 2.2 Data collection

#### 2.2.1 COVID-19 cases

The cases from January 1, 2020 to December 31, 2021 were obtained from the National Notifiable Infectious Diseases Reporting Information System (NNIDRIS) at Guangdong Provincial Center for Disease Control and Prevention.

#### 2.2.2 NPIs and emergency response periods grading

The NPIs were collected from the official website and media reports including cases-based measures (quarantine and isolation), community measures (wearing masks, enhancing personal hand hygiene, and school closures), and travel-related measures. The government implemented Level 1 emergency response on January 23, 2020, after Guangdong Province reported the first case of COVID-19 on January 14, 2020. And then a series of NPIs such as canceling large-scale mass events, wearing face masks in public, and extending school holidays were taken.^1^ According to the study of Xiao et al. (13), we divided the period of 2020–2021 into four periods. Level 1 emergency response was weeks 4–8 in 2020, Level 2 was weeks 9–19, Level 3 was weeks 20–53 in 2020 and the whole of 2021 was divided into the Level 4 emergency response. The higher response level, the stricter the NPIs implemented.

#### 2.2.3 HFMD cases

Data of daily HFMD cases aggregated for each of the 21 cities in Guangdong from January 1, 2012, to December 31, 2021, were obtained from the NNIDRIS. For this study, these daily HFMD cases were first organized into weekly data, and then categorized into different subgroups according to gender (male or female), age (0–2 years, 3–5 years, or 6–14 years), and occupation type (scattered children, kindergarten children or student).

#### 2.2.4 HFMD etiological data

The data were collected from the Guangdong Province Acute Infectious Disease surveillance Information System from January 1, 2012, to December 31, 2021. We first divided the test results of the samples into negative, enterovirus A71 (EV-A71), coxsackievirus A16 (CV-A16) and others, and calculated the weekly total number of cases for each. Then, we calculated the composition ratio of EV-A71 cases and CV-A16 cases to the weekly total.

#### 2.2.5 Meteorological data

Daily meteorological data were obtained from The China Meteorological Data Service Centre.^2^ If there is no meteorological monitoring station in the administrative region of a city, the meteorological monitoring station data of the nearest neighboring city will be used.

#### 2.2.6 Sociodemographic and economic data

Population data for children aged 0–14, and *per capita* GDP for 21 cities were collected from the Guangdong Statistics Yearbook (see Footnote 1).

### 2.3 Statistical analysis

We used the Bayesian structural time series (BSTS) model to construct the counterfactual time series in 2020–2021, and compare the weekly counterfactual cases with observed cases which were further stratified by emergency response periods, population, and cities to evaluate the effects of NPIs on HFMD.

The BSTS model consists of the spike-and-slab method, Kalman filter, and Bayesian model averaging, and was specified as follows:


(1) $$y_{t}={Z_{t}}^{T}\alpha_{t}+\mu_{t}+\tau_{t}+\varepsilon_{t}\;\varepsilon_{t}\sim N\;\left(0,H_{t}\right)\;$$

(2) $$\;\alpha_{t+1}=T_{t}\alpha_{t}+R_{t}\eta_{t}\;\eta_{t}\sim N\;\left(0,Q_{t}\right)\;$$

Eq. (1) is the observation equation, which connects the monitoring data *y_t_* with the potential d-dimensional state vector *α_t_*. Eq. (2) is the transition equation that governs the change of the state vector *α_t_* over time. In this study, *y_t_* is the number of HFMD cases in a certain week t, *Z_t_* is a d-dimensional output vector, *μ_t_* is the control variable (weekly average temperature, weekly average relative humidity, and the weekly composition ratio of CV-A16). Some studies showed that the prevalence of HFMD is affected by meteorological parameters (23–26), which suggests temperature and relative humidity are significantly associated with HFMD incidence. Studies also found that increasing CV-A16 epidemic activity was observed recently in Guangdong (19). Hence, we incorporated them into the BSTS model. *τ_t_* is the time trend control variable, and *ε_t_* is the monitoring error. *Tt*. is a transformation matrix of d*d, *R_t_* is a control matrix of controlling d*q, and *η_t_* is a q-dimensional systematic error. The above state space equation integrates the linear regression, the seasonal trend, and the regression coefficients of covariates over the same period of the number of HFMD cases per week before the intervention, and the Bayesian prior distribution is used for fitting in the analysis process.

First, we fit data from 2012 to 2019 to evaluate the fit of the BSTS model. The data from 2012 to 2017 were used as training data, and the data from 2018 to 2019 were used to simulate. We evaluated the fit of the model using the Mean Absolute Percentage Error (MAPE) or *R*^2^ values, with lower MAPE values indicating better performance and higher *R*^2^ values indicating a better model fit. Then based on the MAPE or *R*^2^, the model has adjusted appropriately so that the accuracy of the model could meet the expected standard. Finally, we used the adjusted model to estimate the weekly counterfactual HFMD cases and evaluate the effects of NPIs on HFMD by comparing the predicted and observed cases from January 20, 2020 (week 4), to December 31, 2021 (week 53), taking into account seasonality and long-term trends in Guangdong Province. The formula is as follows:


(3) $$E^{total}=\frac{\sum_{a}^{\tau }\left[Y\begin{array}{c} c=0\\ a \end{array}\left(t\right)-Y\begin{array}{c} c=1\\ a \end{array}\left(t\right)\right]}{\sum_{a=1}^{\tau }Y\begin{array}{c} c=0\\ a \end{array}\left(t\right)}$$

In Eq. (3), $Y\begin{array}{c} c=0\\ a \end{array}\left(t\right)$ represents the counterfactual HFMD cases in group a at week t and c = 0 represents the counterfactual HFMD cases. $Y\begin{array}{c} c=1\\ a \end{array}\left(t\right)$ represents the number of observed cases in age group a at week t and c = 1 represents the observed cases. τ is the total age group. $E^{\mathrm{total}}$ represents the magnitude of the reduction of the observed cases compared with the expected. We used the indicator “relative reduction” instead of $E^{\mathrm{total}}$ to represent the effects. In addition, we estimated the relative reduction by gender, age, and occupation groups.

Furthermore, to explore the relationship between the relative reduction with COVID-19 incidence and socioeconomic status, we did a Spearman correlation analysis of the relative reduction with the number of COVID-19 cases, COVID-19 incidence, GDP *per capita*, and composition ratio of children aged 0–14 in 2020 and 2021, respectively.

In this study, a value of *p* of <0.05 was considered significant. We implemented the BSTS models using the “*bsts*” and “*CausalImpact*” packages in R soft (version 4.2.1).

## 3 Results

### 3.1 Epidemic of COVID-19 and implementation of NPIs

**Figure 1:**
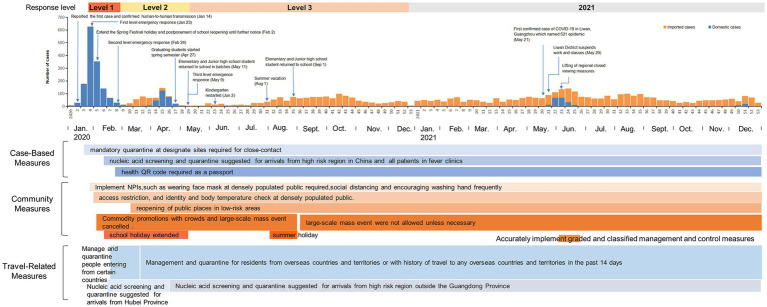
Epidemiological profile of COVID-19 and implementation of synchronous NPIs in Guangdong Province, China, 2020–2021.

The epidemic curve was constructed according to the number of COVID-19 cases per week and the NPIs initiated at different times were also annotated in the Figure 1. We classified NPIs into three categories: case-based measures, community measures, and travel-related measures.

### 3.2 Epidemiological trend of HFMD

The epidemiological trend of HFMD during 2012–2021 was presented in Supplementary Figure S2. We found the HFMD has seasonality and periodicity, with two peaks per year, and the incidence remained high until 2020. The major peak is in May–July and the smaller peak is in September–October.

Table 1 showed the cases and incidence of HFMD by gender, age, and occupation group. Overall, Guangdong reported 59,264 and 291,953 cases in 2020 and 2021, respectively, which was lower than the annual cases in 2012–2019. The incidence was 2.50/1000 in 2020 and was 12.29/1000 in 2021 which was lower than the annual incidence in 2012–2019. We found that the incidence in male was higher than in female. Among the age groups, the HFMD incidence in 2020 and 2021 was lower than the annual incidence of 2012–2019. The incidence in children aged 0–2 and 3–5 years was significantly higher than in those aged 6–14 years. Furthermore, the proportion of scattered children was the most.

**Table 1:** The comparison of HFMD incidence in Guangdong Province in 2012–2019, 2020, and 2021.

Group	2012–2019		2020		2021	
	Average annual cases	Average annual incidence (1/1000)	Cases	Incidence (1/1000)	Cases	Incidence (1/1000)
**Overall**	374,399	20.03	59,264	2.50	291,953	12.29
**Gender**						
Male	228,439	22.31	33,765	2.65	171,684	13.47
Female	145,959	17.27	25,499	2.32	120,269	10.93
**Age**						
0–2 years	239,051	56.69	35,201	8.02	141,818	32.31
3–5 years	115,086	28.93	20,977	4.00	124,644	23.77
6–14 years	20,261	1.93	3,086	0.22	25,491	1.81
**Occupation**						
Scattered children	79%		70%		59%	
Kindergarten children	18%		26%		35%	
Student	3%		4%		6%	

*The incidence is calculated for the gender and age group, and the composition ratio is calculated for the occupation group.

### 3.3 The impact of the NPIs

**Figure 2:**
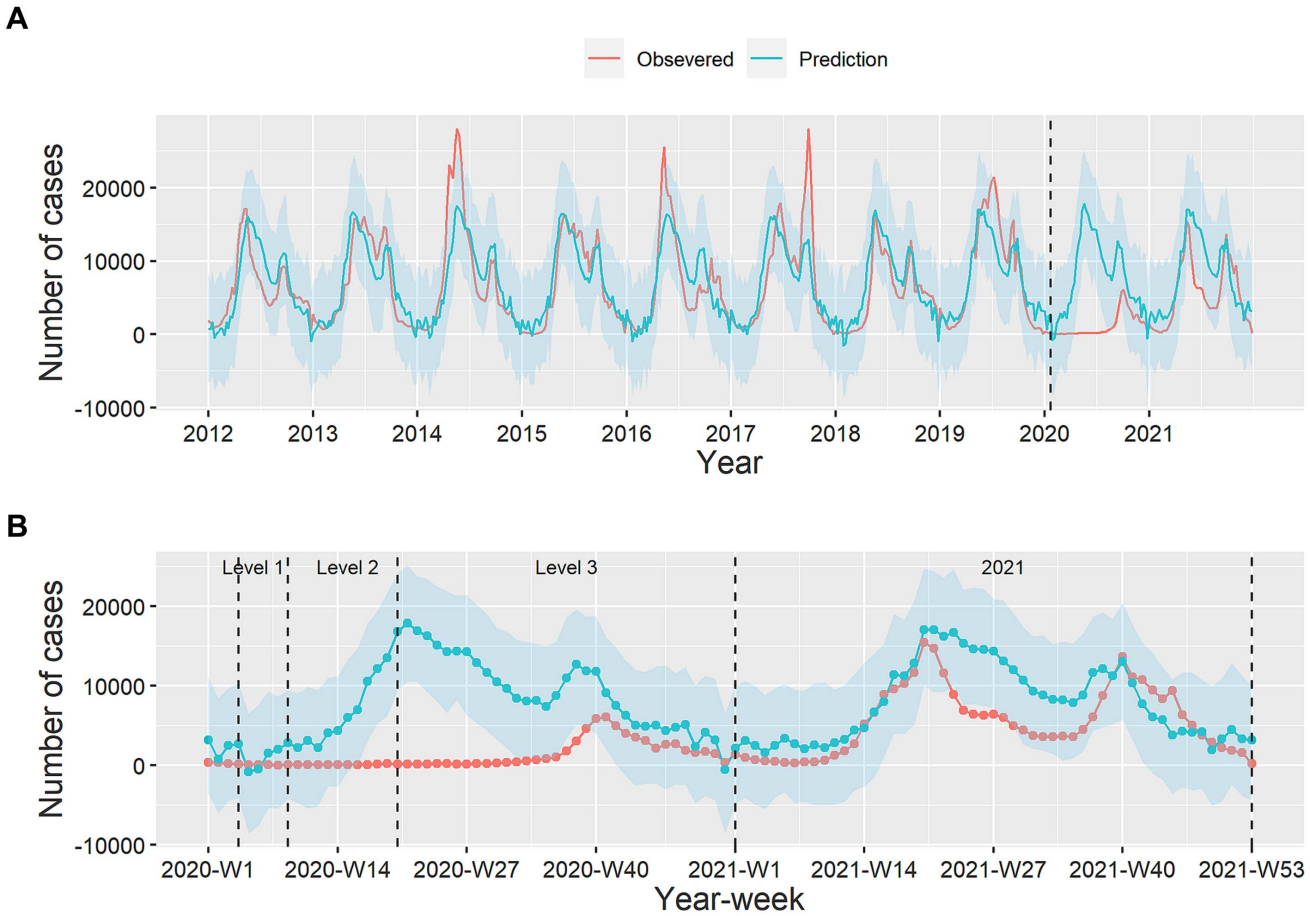
The weekly counts of observed and predicted HFMD cases. Plot **(A)** is the weekly time-series of observed and predicted HFMD cases during 2012–2021, and plot **(B)** is the comparison of observed HFMD cases with prediction in the 4 periods. Red line: observed cases, blue line: predicted cases, blue shadow: 95% confidence interval for prediction.

Figure 2 and Table 2 showed the comparison of the observed and predicted HFMD cases by week during 2020–2021. We found that the observed cases were lower than the predicted counterfactual cases in 2020–2021. In addition, the major peak disappeared and the smaller peak was delayed in 2020. The BSTS model showed that the relative reduction of HFMD cases in Level 1 was 91.10% (95% CI: -∞–97.95%), corresponding to 4,537 averted cases. The relative reduction in Level 2 was 98.28% (95% CI: 97.18–98.74%), averting 67,011 cases. At Level 3, the relative reduction was 82.00% (95% CI: 78.34–84.59%) averting 262,664 cases. While the relative reduction had declined to 29.49% (95% CI: 15.26–39.54%) in 2021, corresponding to 121,115 cases averted (Table 2).

**Table 2:** The relative reduction of HFMD incidence during four periods in Guangdong Province in 2020–2021.

Period	Observed	Predicted (95%CI)	Averted cases	Relative reduction (95%CI)
Level 1 (weeks 4–8 in 2020)	443	4,980 (0–21,644)	4,537	91.10% (−∞–97.95%)*
Level 2 (weeks 9–19 in 2020)	1,175	68,186 (41702–93,595)	67,011	98.28% (97.18–98.74%)
Level 3 (weeks 20–53 in 2020)	57,646	320,310 (266118–374,120)	262,664	82.00% (78.34–84.59%)
Level 4 (2021)	291,953	414,068 (344522–482,910)	121,115	29.49% (15.26–39.54%)
Overall (2020–2021)	351,217	807,544 (708750–905,412)	455,327	56.38% (50.45–61.21%)

^*^For certain period, the lower limits of the predicted become infinitely small because they involved zero predicted cases.

### 3.4 The impact of NPIs by group

**Figure 3:**
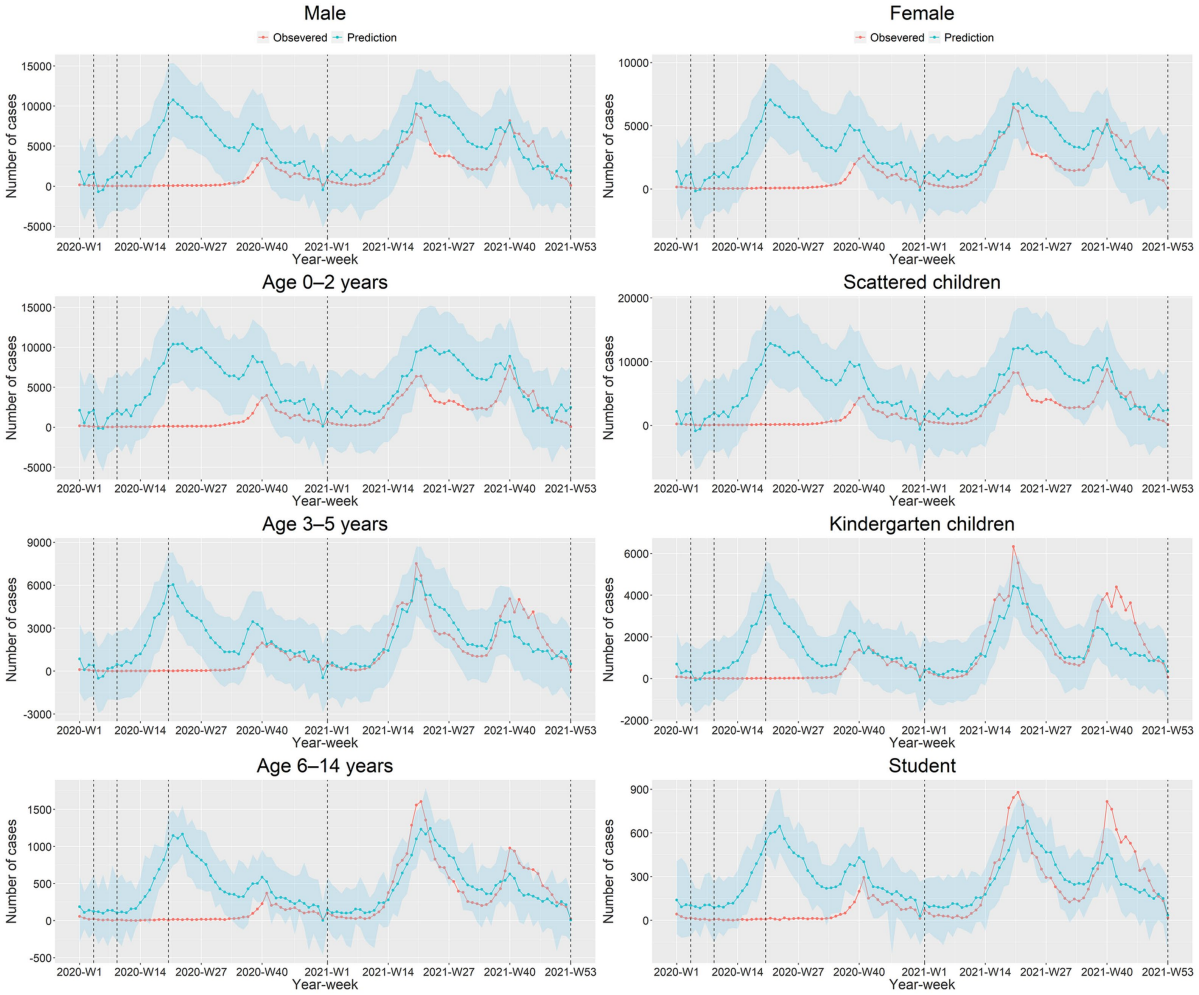
The observed and predicted HFMD cases by gender, age, and occupation during 2020–2021 in Guangdong Province.

Figure 3 and Supplementary Table S1 presented the relative reduction of HFMD cases by gender, age, and occupation during 2020–2021. The relative reduction in male was similar to that in female. In all three age groups, the greatest relative reduction was at Level 2, with 97.88% (95% CI: 96.65–98.45%), 99.12% (95% CI: 98.25–99.41%), and 97.56% (95% CI: 95.35–98.33%), respectively. The relative reduction of 0–2 years was larger than that of 3–5 years and 6–14 years during Level 1–Level 4 except Level 2. The counterfactual HFMD cases in 2021 in children aged 3–5 years and 6–14 years were higher than the observed cases. The situation in different occupations was similar to that in age group, with the greatest relative reduction in both cases at Level 2, which was 97.79% (95% CI: 96.30–98.44%), 99.35% (95% CI: 99.13–99.68%), and 97.55% (95% CI: 96.28–98.20%), respectively. The relative reduction among kindergarten children was higher than scattered children and student in Level 1 and Level 2, whereas that of scattered children was larger than that of kindergarten children and students during Level 3 to Level 4 period. The observed cases of kindergarten children and student were more than predicted in 2021.

### 3.5 The impact of the NPIs by city

Supplementary Figure S3 presented the comparison between observed and predicted HFMD cases in 21 cities by week. We noticed that the observed cases were lower than predicted in 2020, and the major peak disappeared and the smaller peak appeared later in each city. However, the observed cases were higher than predicted at the smaller peak of HFMD prevalence in some cities in 2021, such as Guangzhou and Foshan.

### 3.6 Correlation analysis

As shown in Supplementary Figures S4A,B were the relative reduction in 21 cities in Guangdong Province in 2020 and 2021, respectively. We found that the relative reduction was even larger in non-Pearl River Delta region. Supplementary Figures S4C,D represent the correlation analysis of relative reduction with log COVID-19 cases, *per capita* GDP, the incidence of COVID-19 (1/100000), and composition ratio of children aged 0–14 years in 2020 and 2021, respectively. The relative reduction was positively related (*ρ* = 0.61) to the composition ratio of children aged 0–14years in 2020. The results showed a negative correlation between the relative reduction in log COVID-19 cases (*ρ* = −0.49) and the incidence of COVID-19 (1/100000) in 2021 (*ρ* = −0.49).

## 4 Discussion

In this study, we observed that HFMD incidence in Guangdong decreased dramatically after the implementation of strict COVID-19 NPIs in 2020, and increased slightly in 2021 with certain relaxing of NPIs. The reduction of HFMD varied by periods and populations, and the effect was associated with COVID-19 incidence. This study has provided evidence that the implementation of NPIs is effective in mitigating the transmission of HFMD and that the stricter the NPIs, the greater the reduction. The results would help to develop the prevention strategy for the HFMD epidemic.

### 4.1 Impact of the NPIs

The characteristics of the epidemic have changed in 2020. HFMD in Guangdong province reached its lowest level in a decade due to the strict NPIs against COVID-19. The observed cases were significantly lower than the counterfactual predicted cases. The major peak disappeared, with only one epidemic peak in the autumn. The results were consistent with the findings of previous studies (27, 28). Niu et al. found that the effective reproduction number of HFMD decreased to 0 after the implementation of NPIs (27). These measures could reduce the transmission capacity of HFMD. However, with the decreased of stringency of NPIs, the number of HFMD cases gradually rose and returned to a higher level in 2021.

The relative reduction of HFMD cases was 56.38% (95%CI: 50.45–61.21%) in 2020–2021, which was lower than in the Xi’an study (12). We observed that the implementation of NPIs had a significant effect on HFMD in Guangdong, China, especially in 2020 when the interventions were more stringent. This finding was similar to that of Zhao et al. (11) and Li et al. (12), who found a remarkable decrease in HFMD cases since the implementation of NPIs in 2020. The main modes of transmission of HFMD include fecal-oral transmission, respiratory transmission and contact transmission (29, 30). And during the COVID-19 epidemic, some NPIs such as mask wearing, frequent hand washing and social distancing restrictions were implemented, which reduced the transmission of HFMD (31, 32). This suggested that developing good hygiene habits can effectively reduce HFMD, which needs attention from schools and parents. The largest reductions were in Level 1 and 2 periods, and the smallest in 2021, at 29.49%. Large-scale school closures occurred in Level 1 and 2 periods, while school closures only applied in epidemic areas during specific periods in 2021. This indicates that school closure may be an important NPI to control HFMD (33). Kindergartens are the common sites for HFMD outbreaks for they are the most socially dense environments (34–37), making it easy for HFMD to spread among children. A previous study showed that school closure reduced transmission by more than 50% (38). Hence, it is necessary for the government to consider closing schools to contain the outbreak. The largest reduction was at Level 2 rather than Level 1, possibly because of the delayed effect of the NPIs on HFMD transmission (13). Our results provided evidence that the implementation of stricter NPIs would provide better protection for children aged 0–14 years old, and suggested that it is important for schools and parents to develop good hygiene habits in children.

### 4.2 Impact of the NPIs by gender, age, and occupation

We observed that the relative reduction in male was similar to that in female. In terms of age groups, the best protection was observed in the 0–2 years group, whose highest reduction was 97.88% (96.65–98.45%) in Level 2. Our study suggested that NPIs such as home isolation can significantly reduce the cases of children under 2 years old. Previous studies have shown that children under 2 years old were more likely to be infected with HFMD, so strengthening the protection of children under 2 years old may be the key issue for the HFMD control (33). Furthermore, we found that the observed cases in children aged 3–5 and 6–14 years old were higher than expected in 2021. One possible reason for this shift may be that, with the under-controlled epidemic and relaxing NPIs, people participated in more economic and social activities (39, 40), which increased the interpersonal contacts and the risk of HFMD transmission (41, 42). In terms of occupation groups, the largest reduction occurred in Level 1 or Level 2, and the kindergarten children and student rebounded in 2021. It may be associated with the school reopen (11, 43). Luca et al. showed that school closure can reduce the spread of infectious diseases, but it would obtain a rebound effect when schools reopened after the closure (44). As the interventions against COVID-19 are relaxed and children return to school, the HFMD epidemic is likely to rise to higher levels. It is important for the government to increase the health knowledge of parents about HFMD (45) and adopt some NPIs (such as home isolation and school closure) to control it. In addition, some virus showed periodicity on the scale of 1 or 2 years (46). The rebound in 2021 may be related to the changes in the type of virus circulating. In conclusion, the effect was greater for 0–2 years than for others. If necessary, school closure may be effective in controlling the HFMD epidemics.

### 4.3 Impact of the NPIs on different regions

The observed HFMD cases decreased to varying degrees compared with the counterfactual cases in 21 cities in 2020. This may be related to the stringency of the implementation effects of the NPIs in different regions. HFMD rebounded mainly in the Pearl River Delta region in 2021, which may be related to the high mobility of the population and more interpersonal contact with people (42) after the relaxation of the implementation of NPIs (12).

The correlation analysis showed that the reduction of HFMD was positively related to the proportion of children aged 0–14 in 2020. The implementation of NPIs has prevented children from being exposed to HFMD virus, resulting in the decrease in the infection (28). Furthermore, our study showed that the reduction of HFMD was negatively related to the COVID-19 cases and incidence in 2021, meaning that regions with more stringent NPIs have fewer COVID-19 cases and a greater reduction in HFMD.

### 4.4 The strength and limitations

Using multi-source data from 21 cities in Guangdong Province from 2012 to 2021, we quantitatively assessed the effects of COVID-19 NPIs on HFMD. In addition, we incorporated influencing factors such as aetiological and meteorological data into the model to provide more reliable results. Our study has several limitations. First, HFMD cases may have been underdiagnosed and underreported to some extent in 2020–2021 due to the fear of COVID-19 infection preventing people from going to a hospital, or some medical institutions may not operate regularly. Second, this study is an ecological research design, the direct effect of a specific non-pharmaceutical intervention on HFMD needs further study. Third, the effect of EV-A71 vaccination was not considered in this study. Previous studies (47, 48) have shown that the EV-A71 vaccine provides good protection against EV-A71-associated HFMD, while showing limited effectiveness against non-EV-A71-associated HFMD. Additionally, one study also (49) found that the overall incidence of HFMD did not decrease after EV-A71 vaccination, although the EV-A71-associated HFMD cases decreased significantly. The EV-A71 vaccination may have a small effect on the overall incidence, so the impact of vaccination on the prevalence of HFMD was not considered in this study. Finally, there may be some uncertainty in estimating the reduction in cases of HFMD due to different periods of school closure.

## 5 Conclusion

This study demonstrated the effects of NPIs in reducing HFMD cases during COVID-19 epidemic in Guangdong Province, which varied in different intervention periods, populations and regions. Our study suggests that timely school closures are an effective way to prevent the HFMD outbreak, and NPIs such as hand washing and mask-wearing would significantly reduce the transmission of HFMD. This study has provided a vivid case to the response of HFMD and these findings will help the policy makers in the prevention and control of HFMD.

## Data availability statement

The data analyzed in this study is subject to the following licenses/restrictions: the dataset used in the study is not public available, while it was available from the corresponding author. Requests to access these datasets should be directed to JPX, jpengx@163.com; MZ, 409782078@qq.com.

## Ethics statement

The studies involving humans were approved by the Ethics Committee of the Guangdong Provincial Center for Disease Control and Prevention (No. W96-027 E-202104). The studies were conducted in accordance with the local legislation and institutional requirements. Written informed consent for participation was not required from the participants or the participants’ legal guardians/next of kin in accordance with the national legislation and institutional requirements. Written informed consent was obtained from the individual(s), and minor(s)’ legal guardian/next of kin, for the publication of any potentially identifiable images or data included in this article.

## Author contributions

LZ: Data curation, Formal analysis, Methodology, Visualization, Writing – original draft. FY: Data curation, Formal analysis, Visualization, Writing – original draft. ZZ: Writing – review & editing. WLZ: Writing – review & editing. ZR: Writing – review & editing. JH: Formal analysis, Visualization, Writing – review & editing. XL: Writing – review & editing. JZ: Methodology, Writing – review & editing. BZ: Writing – review & editing. YHL: Data curation, Visualization, Writing – review & editing. YQ: Writing – review & editing. QZ: Visualization, Writing – review & editing. ZH: Visualization, Writing – review & editing. YYL: Data curation, Writing – review & editing. XH: Writing – review & editing. WYZ: Writing – review & editing. JQX: Writing – review & editing. YL: Funding acquisition, Writing – review & editing. QC: Writing – review & editing. JPX: Data curation, Funding acquisition, Methodology, Resources, Writing – review & editing. MZ: Data curation, Resources, Writing – review & editing.

## Footnotes

^1^ https://www.gd.gov.cn/

^2^ http://www.cma.gov.cn/
