# Supplementary material for: Assessing the impact of COVID-19 interventions on the hand, foot and mouth disease in Guangdong Province, China: a Bayesian modeling study
Source: Front Public Health. 2024 Jan 11;11:1307321. doi: 10.3389/fpubh.2023.1307321 (PMC10860754; doi:10.3389/fpubh.2023.1307321)
Supplement: Supplementary file 1 [file Data_Sheet_1.docx]

**Supplementary materials**

**Assessing the impact of COVID-19 interventions on the hand, foot and mouth disease in Guangdong Province, China: a Bayesian modeling study**

**Figure S1** Map of 21 cities and population distribution in Guangdong Province in 2020

**Figure S2** Epidemiological trend of HFMD in 2012–2021 by year-month (A), year (B), and month (C)

**Figure S3** The observed and predicted HFMD cases among 21 cities during 2020–2021 in Guangdong Province

**Figure S4** The relative reduction of 21 cities in Guangdong Province and the correlation analysis. (A and B are the spatial distribution of the relative reduction in the 21 cities in 2020 and 2021, respectively. C and D are the relative reduction with log COVID-19 cases, per capita GDP, the incidence of COVID-19 (1/100000), and the composition ratio of children aged 0–14 in 2020 and 2021, respectively)

**Table S1** The relative reduction of gender, age and occupation groups in 2012–2021

**
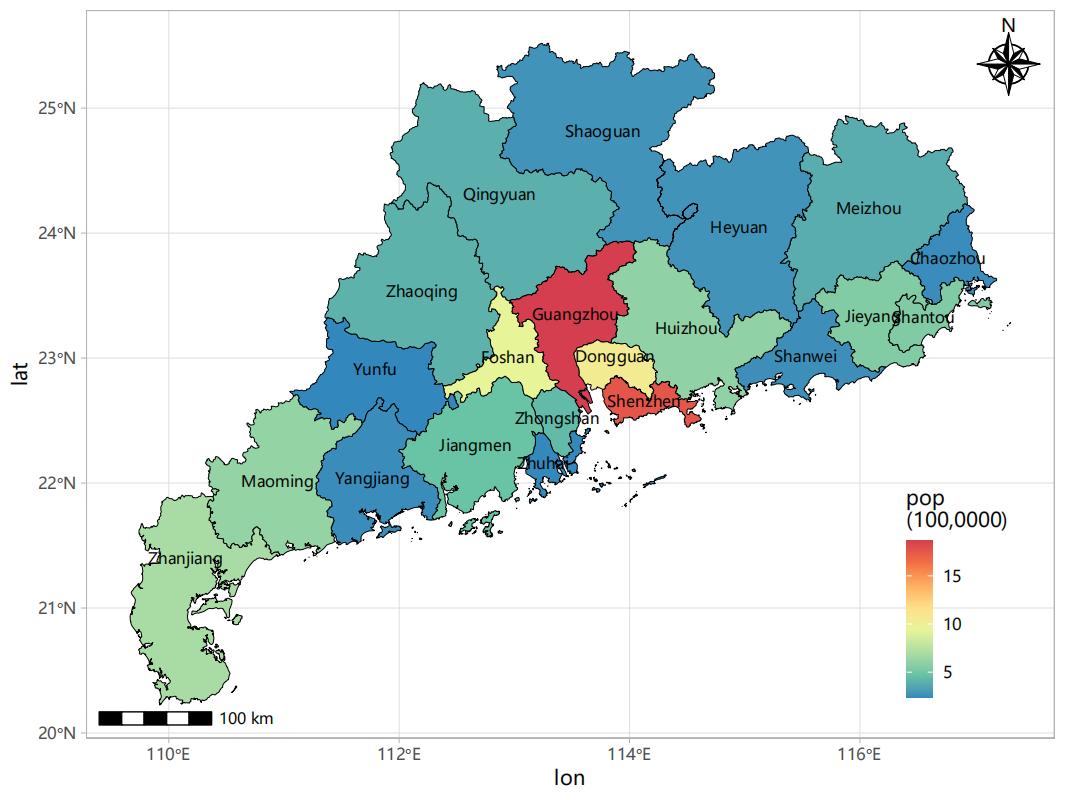
Figure S1** Map of 21 cities and population distribution in Guangdong Province in 2020


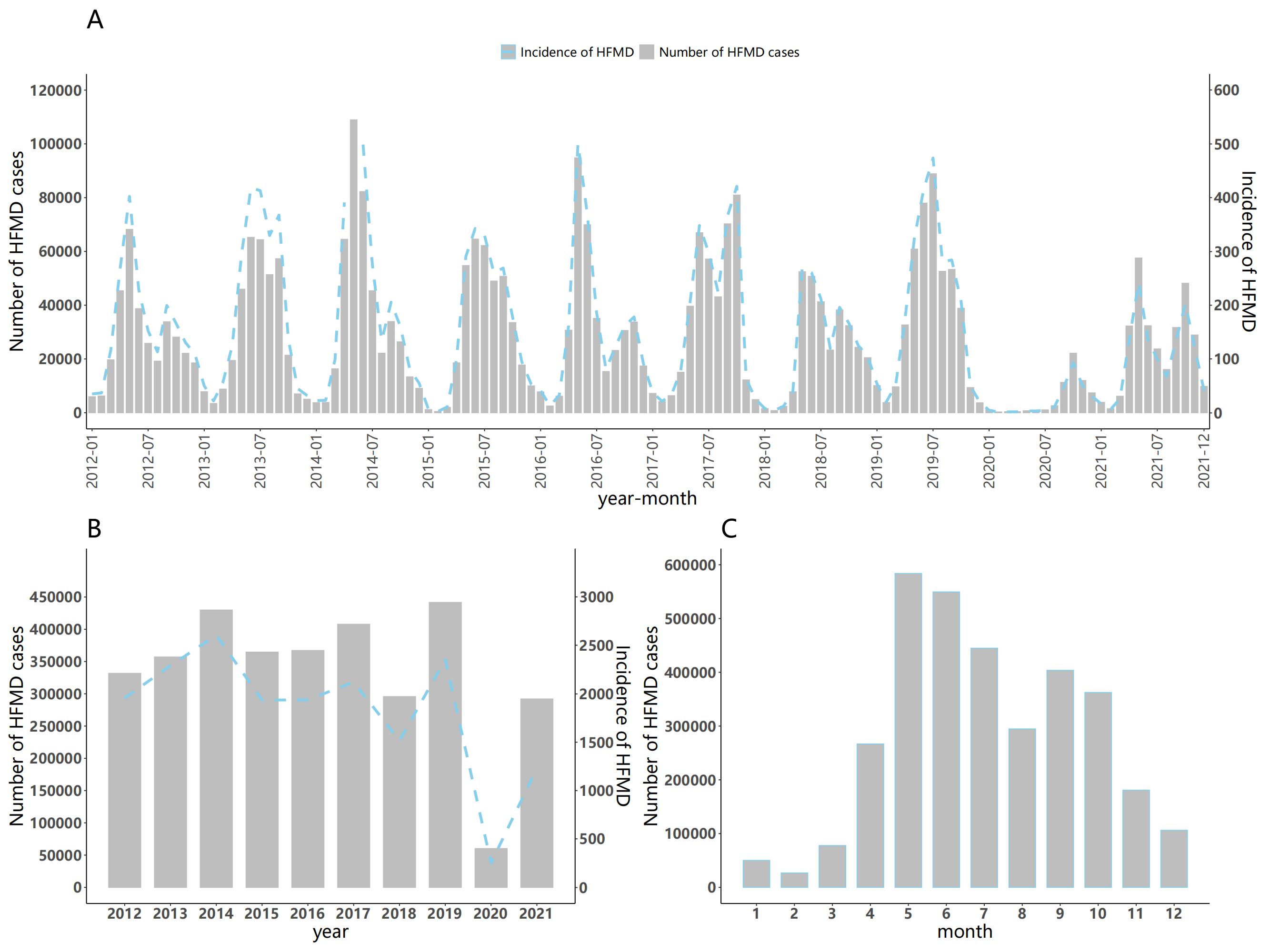


**Figure S2** Epidemiological trend of HFMD in 2012–2021 by year-month (A), year (B), and month (C)


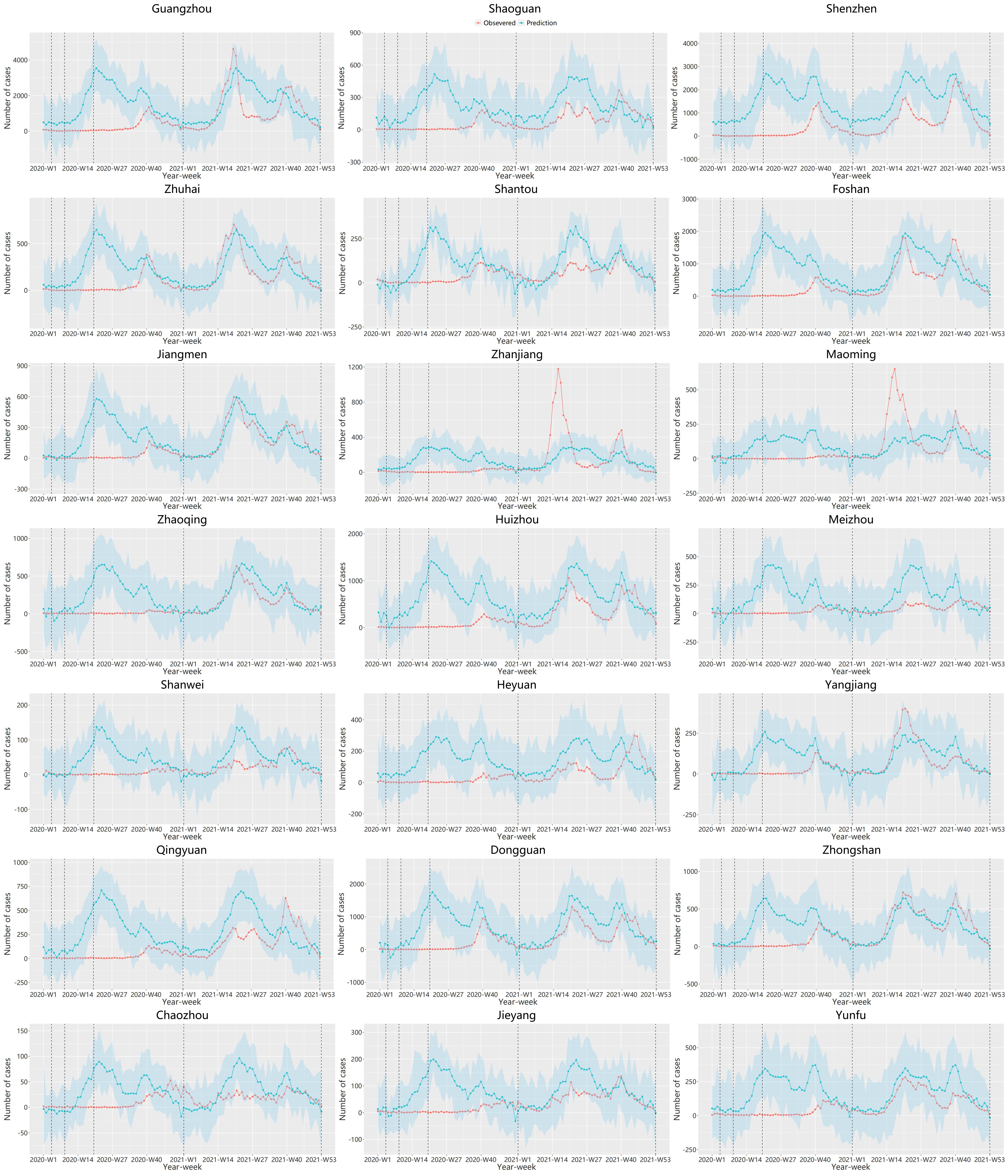


**Figure S3** The observed and predicted HFMD cases among 21 cities during 2020–2021 in Guangdong Province


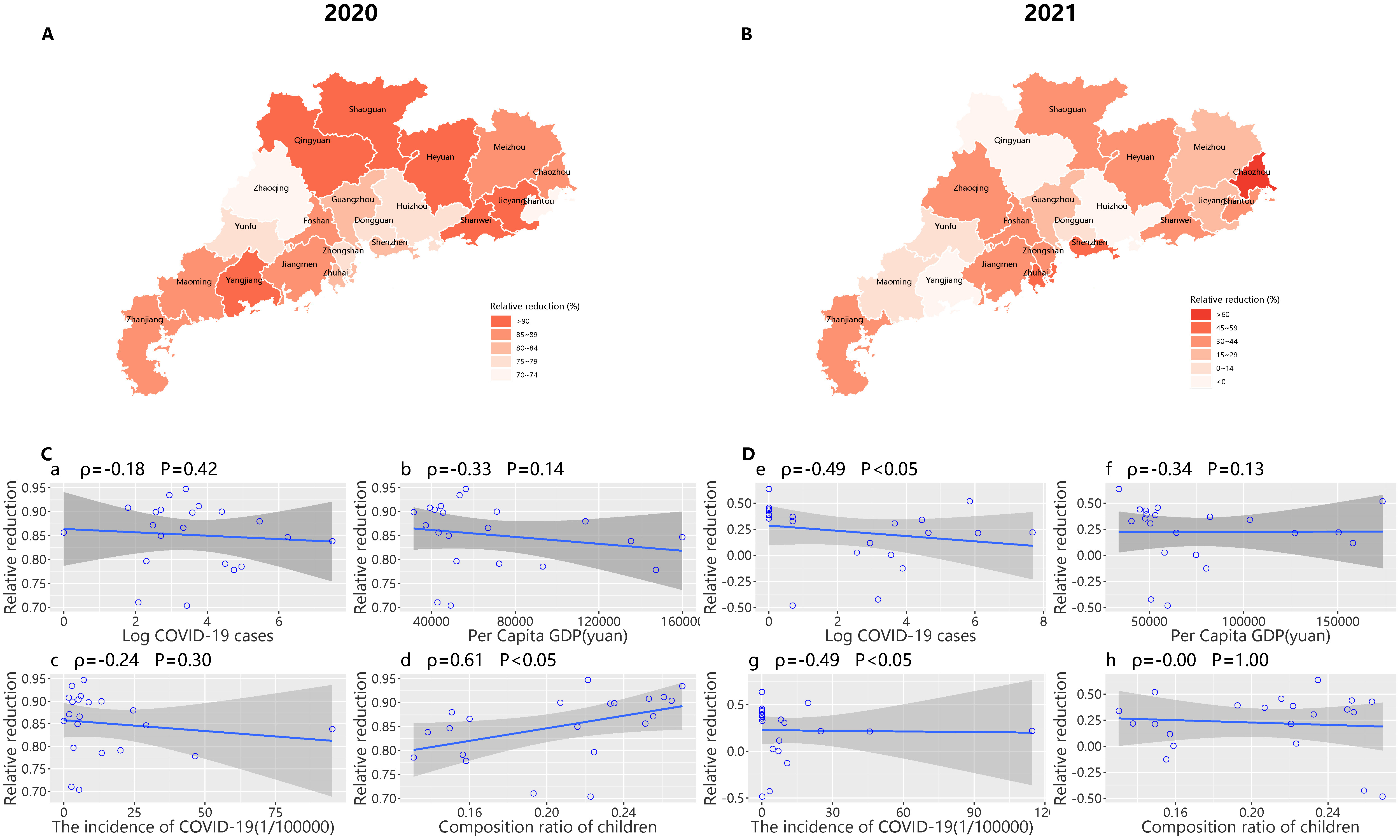


**Figure S4** The relative reduction of 21 cities in Guangdong Province and the correlation analysis. (A and B are the spatial distribution of the relative reduction in the 21 cities in 2020 and 2021, respectively. C and D are the relative reduction with log COVID-19 cases, per capita GDP, the incidence of COVID-19 (1/100000), and the composition ratio of children aged 0–14 in 2020 and 2021, respectively).

**Supplementary Table S1** The relative reduction of gender, age and occupation groups from 2020 to 2021

| **Group** | **Level 1 (weeks 4–8 in 2020)** | | **Level 2 (weeks 9–19 in 2020)** | | **Level 3 (weeks 20–53 in 2020)** | | **Level 4(2021)** | |
| --- | --- | --- | --- | --- | --- | --- | --- | --- |
|  | Observed | Relative reduction  (95%CI) | Observed | Relative reduction  (95%CI) | Observed | Relative reduction  (95%CI) | Observed | Relative reduction  (95%CI) |
| **Gender** |  |  |  |  |  |  |  |  |
| Male | 245 | 89.63%  (-∞–98.02%)**^*^** | 589 | 98.54% (97.56%–98.95%) | 32931 | 82.87% (79.37%–85.36%) | 171684 | 30.63% (16.30%– 40.70%) |
| Female | 198 | 92.24%  (-∞–97.78%)**^*^** | 586 | 97.87% (96.58%–98.45%) | 24715 | 80.72% (77.00%–83.44%) | 120269 | 27.62% (13.28%–37.66%) |
| **Age** |  |  |  |  |  |  |  |  |
| 0**–**2 years | 290 | 93.75%  (-∞–98.14%)**^*^** | 898 | 97.88% (96.65%–98.45%) | 34013 | 84.67% (81.85%–86.69%) | 141818 | 47.15% (37.04%–54.55%) |
| 3**–**5 years | 95 | 88.69%  (-∞–98.23%)**^*^** | 186 | 99.12% (98.25%–99.41%) | 20696 | 74.74% (67.85%–79.28%) | 12464 | -3.45% (-28.78%–13.81%) |
| 6**–**14 years | 58 | 90.66%  (-∞–95.45%)**^*^** | 91 | 97.56% (95.35%–98.33%) | 2937 | 83.27% (78.84%–85.98%) | 25491 | -3.79% (-29.15%–13.50%) |
| **Occupation** |  |  |  |  |  |  |  |  |
| Scattered children | 352 | 88.27%  (-∞–97.82%)**^*^** | 1049 | 97.79% (96.30%–98.44%) | 39881 | 84.09% (80.85%–86.37%) | 171737 | 45.36% (33.95%–53.46%) |
| Kindergarten children | 47 | 93.71%  (-∞–98.83%)**^*^** | 70 | 99.35% (99.13%–99.68%) | 15508 | 70.35% (62.06%–75.60%) | 103741 | -24.82% (-53.85%– -4.65%) |
| Student | 44 | 91.02%  (-∞–95.27%)**^*^** | 56 | 97.55% (96.28%–98.20%) | 2257 | 79.36% (75.17%–82.34%) | 16475 | -9.17% (-30.17%–5.78%) |

^*^ For certain period, the lower limits of the predicted become infinitely small because they involved zero predicted cases.
